# Spatial Patterns and Trends of Summertime Low Cloudiness for the Pacific Northwest, 1996–2017

**DOI:** 10.1029/2020GL088121

**Published:** 2020-08-19

**Authors:** Alex W. Dye, Bharat Rastogi, Rachel E. S. Clemesha, John B. Kim, Roger M. Samelson, Christopher J. Still, A. Park Williams

**Affiliations:** ^1^ Department of Forest Ecosystems and Society Oregon State University Corvallis OR USA; ^2^ USDA Forest Service Corvallis Forestry Sciences Laboratory Pacific Northwest Research Station Corvallis OR USA; ^3^ Cooperative Institute for Research in Environmental Sciences University of Colorado Boulder Boulder CO USA; ^4^ Global Monitoring Laboratory National Oceanic and Atmospheric Administration Boulder CO USA; ^5^ Scripps Institution of Oceanography University of California, San Diego La Jolla CA USA; ^6^ College of Earth, Ocean, and Atmospheric Sciences Oregon State University Corvallis OR USA; ^7^ Lamont‐Doherty Earth Observatory Columbia University Palisades NY USA

**Keywords:** low clouds, Pacific Northwest, GOES, Oregon, Washington, fog

## Abstract

Summertime low clouds are common in the Pacific Northwest (PNW), but spatiotemporal patterns have not been characterized. We show the first maps of low cloudiness for the western PNW and North Pacific Ocean using a 22‐year satellite‐derived record of monthly mean low cloudiness frequency for May through September and supplemented by airport cloud base height observations. Domain‐wide cloudiness peaks in midsummer and is strongest over the Pacific. Empirical orthogonal function (EOF) analysis identified four distinct PNW spatiotemporal modes: oceanic, terrestrial highlands, coastal, and northern coastal. There is a statistically significant trend over the 22‐year record toward reduced low cloudiness in the terrestrial highlands mode, with strongest declines in May and June; however, this decline is not matched in the corresponding airport records. The coastal mode is partly constrained from moving inland by topographic relief and migrates southward in late summer, retaining higher late‐season low cloud frequency than the other areas.

## Key Points

- Summer low cloudiness separates into four distinct spatiotemporal sectors: oceanic, terrestrial highlands, coastal, and northern coastal modes
- Low cloudiness is strongest over the Pacific, where it peaks in midsummer; terrestrial and coastal areas peak in late summer
- Satellite records suggest a terrestrial highlands decline in low cloudiness over time, but airport records do not

## 1 Introduction

Low stratiform clouds (i.e., stratus, stratocumulus, and fog) are a prominent summertime feature in the Pacific Northwest (PNW) region of North America. Characteristics of summertime low clouds have been extensively studied in many west coast environments including North America (Clemesha et al., 2016, 2017; Filonczuk et al., 1995; Iacobellis & Cayan, 2013; Rastogi et al., 2016; Schwartz et al., 2014), the Chilean coast (e.g., Garreaud et al., 2008; McIntyre et al., 2005), and the southwest African coast (e.g., Cermak, 2012; Eckardt et al., 2013). Over the ocean, these clouds typically form in summer‐dry continental west coast climates under a low‐altitude temperature inversion, facilitated by a combination of coastal upwelling of cold water and adiabatic warming of air aloft in the descending branches of the Northern Hemisphere Hadley cell (Lilly, 1968; Wood, 2012). Low clouds also form along the PNW coast when onshore surges of cool, moist marine air are overlain by warm, dry continental air, sometimes decreasing temperatures and increasing cloud cover as far inland as the western Cascade foothills (Leipper, 1994; Mass et al., 1986).

By the end of the 21st century, PNW summers are projected to be drier and hotter (Mote & Salathé, 2010), more closely resembling present‐day northern California (Fitzpatrick & Dunn, 2019). During coastal California's summers, low clouds regulate land surface temperatures (Iacobellis & Cayan, 2013), provide shade and supplemental moisture for native vegetation (Carbone et al., 2013; Fischer et al., 2009; Johnstone & Dawson, 2010; Williams et al., 2008), improve agricultural water use efficiency (Baguskas et al., 2018), reduce wildfire potential (Emery et al., 2018; Williams et al., 2018), maintain healthy stream levels (Sawaske & Freyberg, 2015), and modulate heat waves (Clemesha et al., 2018). Similar effects likely occur in the PNW. These comprehensive impacts necessitate detailed assessments of spatiotemporal characteristics of low clouds in the past, present, and future (Torregrosa et al., 2014). In the future, low clouds could serve either as a buffer mitigating higher temperature and moisture loss or as positive feedbacks to climate change if they are reduced. The latter has been projected to occur in other regions; for example, a regional climate simulation for California projected a long‐term decline in coastal fog (O'Brien et al., 2013), while historical airport observations from northern California suggest a possible decline of summer fog over the twentieth century (Johnstone & Dawson, 2010). And increasing urbanization in southern California has been implicated in a dramatic reduction in summertime stratus cloud frequency in that region (Williams et al., 2015).

Evidence suggests that development of native vegetation in the PNW was facilitated by supplemental moisture inputs provided by low clouds and fog drip (Harr, 1982; Long & Whitlock, 2002). In recent years, coastal PNW Douglas‐fir trees have been hypothesized to be negatively impacted by fog, as spores of Swiss needle cast fungus transported by water droplets and frequent leaf wetting from fog immersion may increase the severity of this disease (e.g., Mildrexler et al., 2019). Drier conditions would increase wildfire vulnerability of some of the highest densities of burnable biomass in North America (Davis et al., 2017), with low cloud shading and moisture inputs potentially tempering the drying of these fuels (Williams et al., 2018). Major PNW cities like Olympia, Portland, Seattle, and Vancouver exceed national averages for population growth (U.S. Census Bureau, 2019; Statistics Canada, 2019), and the role of clouds in moderating urban temperatures will become increasingly important in the future (Williams et al., 2015). Some projections (e.g., Jacox et al., 2015) suggest upwelling of cool coastal water will intensify in the future, which could maintain or even increase coastal low cloudiness.

Despite the importance of coastal low cloud dynamics, they remain one of the more difficult atmospheric phenomena to model (Koračin et al., 2014), precluding definitive statements about current and future impacts. With the exception of Mass et al. (1986), little emphasis has been devoted specifically to the PNW, and long‐term patterns and trends have not yet been characterized. In this paper, we use a 22‐year gridded record of satellite‐derived low cloud frequency to produce the first comprehensive maps and spatiotemporal summary of low cloudiness during PNW summers. Our work identifies key patterns and trends that are a critical foundation for further understanding the current and future role of low clouds in the PNW.

## 2 Data and Methods

### 2.1 GOES Low Cloud Frequency

**Figure 1:**
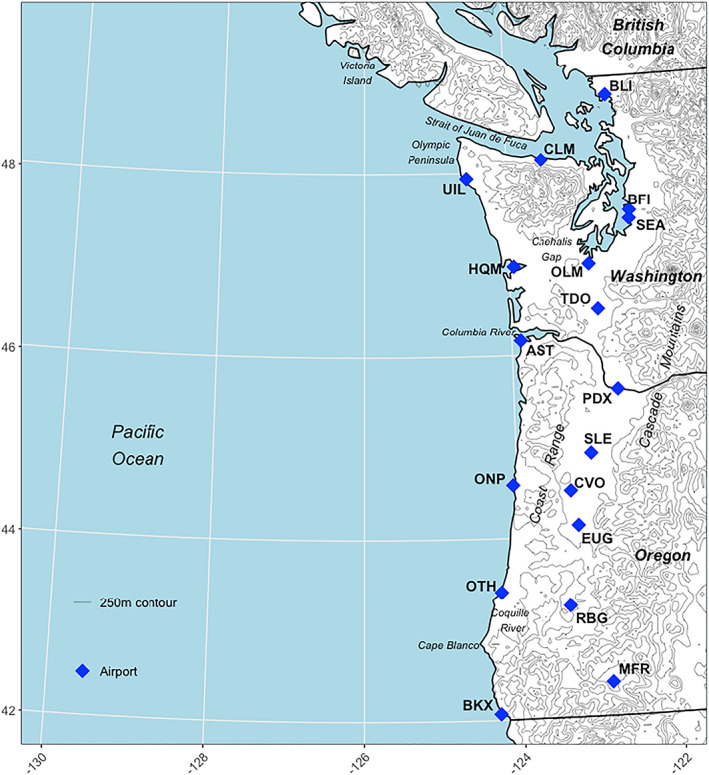
The study domain, with airports used for comparison, key geographic features, and 250‐m contour lines. The standard three‐letter airport codes from the International Air Transport Association (IATA) is used. Full airport names are in Figure 3.

We use the 0.04° × 0.04° gridded coastal low cloud frequency record derived from the National Aeronautics and Space Administration/National Oceanic and Atmospheric Administration (NASA/NOAA) Geostationary Operational Environmental Satellite (GOES) as described in Clemesha et al. (2016). In brief, their algorithm separated low‐level from high‐level clouds and clear sky to identify presence or absence of low clouds at half‐hour increments. Daily low cloud frequency is defined as the fraction of half‐hourly observations with low cloud presence out of the number of total observations for each 24‐hr period. We aggregated all daily observations from Clemesha et al. (2016) to monthly means, for a domain spanning 130°W to 122°W and 42°N to 49.50°N (Figure 1), for May through September from 1996–2017. To limit potential influences of high elevation snowpack on the satellite‐based cloud classification, we removed pixels greater than 1,500 m above sea level, where elevation was derived from the North America Elevation 1‐Km GRID and aggregated to match GOES resolution (USGS and Natural Resources Canada, 2007). The Clemesha et al. (2016) data set was created specifically to identify coastal low clouds influenced by the marine layer, not low clouds formed convectively over land. For this reason, we limited the inland extent of our domain to 122°W; however, this still stretches the original purpose of the data set.

### 2.2 Coastal Airport Low Cloud Frequency

To support results derived from GOES we also examined hourly ceiliometer cloud base height records at 18 airports (Iowa Environmental Mesonet, 2019), selected for representation of the full spatial domain (Figure 1).

We defined daily airport low cloud frequency as the percentage of total hourly observations over each 24‐hr period that met two criteria: cloud cover as “overcast” (>87% cover) or “broken” (50–87% cover) and cloud base height at or below 2,000 m above sea level. We then compared daily averages and annual temporal trends with the GOES‐derived low cloud frequencies averaged across a 3 × 3 pixel (0.12° × 0.12°) area with the airport's point location in the center pixel of the area. The 3 × 3 pixel area was chosen to minimize edge effects where an airport may lie on or near the edge of an original 0.04° × 0.04° pixel.

### 2.3 Identification of Spatiotemporal Patterns and Trends

We implemented an empirical orthogonal function (EOF) analysis in R (R Core Team, 2017) to identify distinct spatiotemporal regions of low cloud frequency. To make calculations computationally efficient and results easier to interpret, we aggregated pixels to a coarser 0.24° × 0.24° resolution; this decision was justified by an exploratory analysis using the original 0.04° × 0.04° data that showed little difference to the regridded results. We removed high‐frequency variability by calculating monthly means and removed the seasonal cycle in mean cloud frequency by calculating anomalies from monthly climatologies. EOF modes were computed from the resulting monthly anomalies (five summer anomalies per year for 22 years), which retain only the interannual variability for each month. The four leading EOF modes contained 91% of total variance and each identified physically interpretable spatial patterns of variability.

We computed the fraction of full PNW‐wide variance, as well as the fraction of within‐cell variance, explained by each mode. The latter distinguishes cases where a mode is locally important, even if it does not explain a high fraction of PNW‐wide variance.

Statistical significance (*α* ≤ 0.05) of the 22‐year trend for each mode was assessed with a nonparametric Mann‐Kendall test (McLeod, 2015) on both monthly and annual (May–September average for each year) EOF amplitudes. Autocorrelation was minimal and not incorporated in the trend tests. Only four series had statistically significant autocorrelations (*α* ≤ 0.05): EOF #2 September, 2‐year lag (autocorrelation = −0.45); EOF #2 Annual, 3‐year lag (0.45); EOF #3 Annual, 3‐year lag (−0.50); and EOF #4 August, 2‐year lag (−0.44). Due to the short 22‐year time frame of our data series, uncertainty in trend magnitude is high, and trends must be interpreted with caution. For the same reason, we did not rigorously explore the spectral properties of the time series of low cloud frequency. We performed an identical set of trend tests on the original GOES‐derived observations at the same 0.24° × 0.24° resolution as the EOF analysis.

## 3 Results

### 3.1 Summertime Progression of Low Cloud Frequency

**Figure 2:**
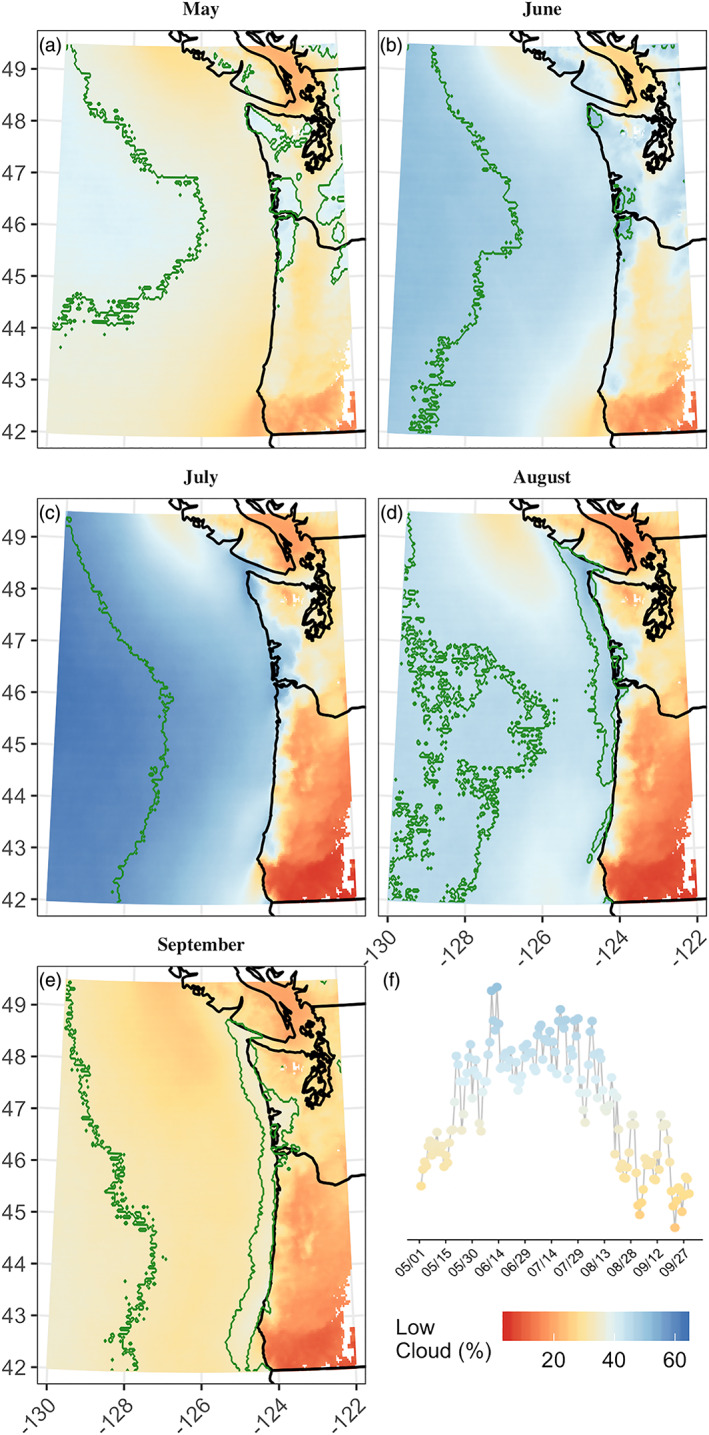
Average 1996–2017 low cloud frequency for 0.04° × 0.04° GOES pixels: (a) May, (b) June, (c) July, (d) August, (e) September, and (f) the domain‐wide daily mean low cloud frequency from 1 May to 30 September. Green contour lines surround the 75th percentile for each month: May—37.71%, June—47.91%, July—58.00%, August—44.96%, and September—32.42%.

The 22‐year mean May through September GOES‐derived low cloudiness shows, on average, persistent low clouds over the Pacific Ocean west of approximately 127°W during all five months and peaking in July (Figure 2). Coastal areas also peak in midsummer but retain more frequent low cloudiness relative to the region into August and September. Despite overall domain‐wide lower frequencies in late summer, by September there is a distinct southward shift in low cloud frequency over the ocean and coastal areas (Figure 2—green contours). Further inland, low cloudiness tends to peak earlier in the season. Inland intrusion of high frequencies of low cloudiness roughly follows the 250 m elevation contour; this topographic constraint is starkly visible in southwest Oregon (around 43°N), where the steep slopes of the southern Coast Range drive a severe contrast from high frequency of low clouds over the coast to low frequency inland. Near this same latitude, coastal Pacific frequencies are consistently lower in all months relative to northern coastal areas, likely the result of clearing from marine layer shallowing in the orographically intensified expansion fan flow around Cape Blanco (Perlin et al., 2004; Pratt & Whitehead, 2007, their Figure 4.3.1).

**Figure 3:**
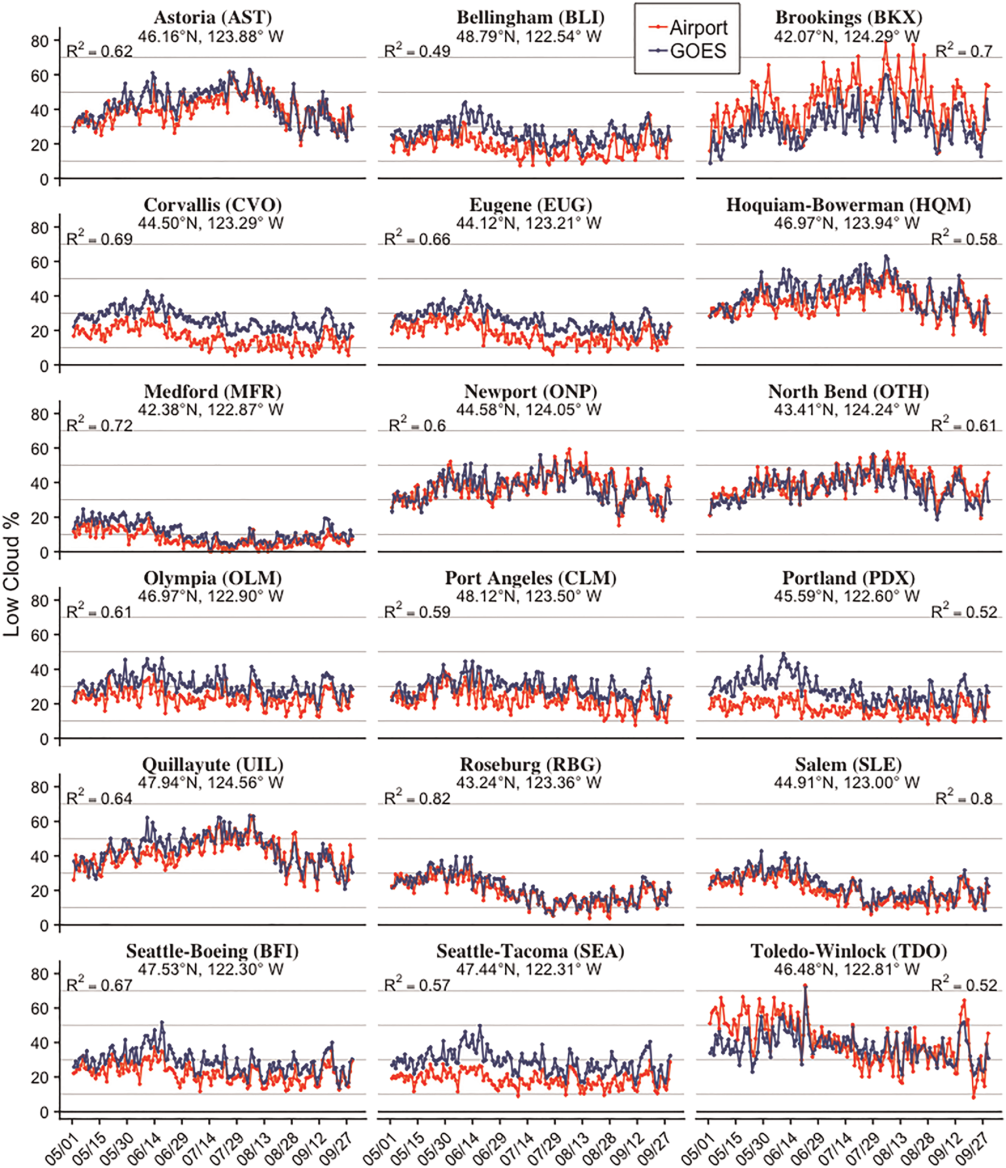
Daily (1 May to 30 September) average low cloud frequency for 18 airports and overlapping 3 × 3 pixel area GOES. Note that airport records were available only after 2005 at Brookings and before 2007 at Toledo‐Winlock and GOES records were also truncated at these airports. *R* ^2^ values for Pearson's correlations between both daily averages are provided.

Seasonal variations of airport records are consistent with GOES‐derived low cloud frequency on a seasonal time scale (Figure 3), displaying the differing characteristics between inland (e.g., Seattle‐Tacoma, Eugene, and Roseburg), where low clouds are overall less frequent and peak in early summer, and coastal locations (e.g., Quillayute, Astoria, and Newport), where low clouds are more frequent and peak in late summer.

### 3.2 EOF #1: Oceanic Mode

**Figure 4:**
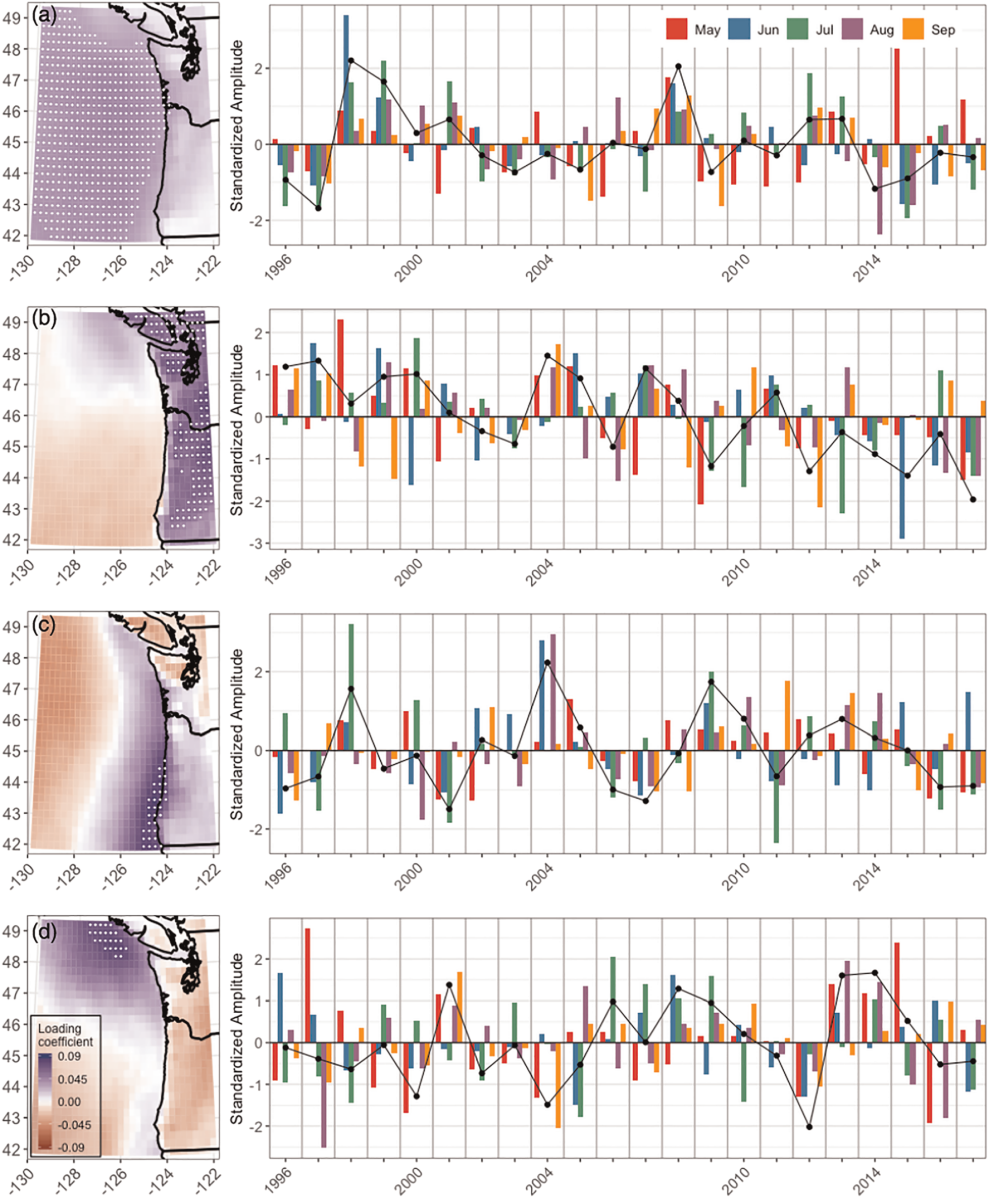
Loading coefficients for 0.24° × 0.24° GOES pixels and standardized amplitude time series for (a) EOF #1 (69.53% of domain‐wide variance), (b) EOF #2 (13.05% of variance), (c) EOF #3 (4.74% of variance), and (d) EOF #4 (2.8% of variance). White dots denote cells that equal or exceed 75% of the maximum within‐mode per‐pixel explained variance: ≥70% for EOF #1, ≥59% for EOF #2, ≥27% for EOF #3, and ≥16% for EOF #4.

Seventy percent of overall PNW‐wide variance is explained by the first EOF mode, loading most strongly west of 126°W and between 43°N and 47°N in the Pacific and also extending inland in low elevation areas (Figure 4a). Low clouds are consistently frequent, exceeding 30% in most months (Figure 2). EOF #1 amplitudes do not exhibit significant temporal trends.

### 3.3 EOF #2: Terrestrial Highlands Mode

EOF #2 (Figure 4b) explains 13% of overall PNW‐wide variance and loads predominantly inland from roughly the 250‐m elevation contour of the coastal mountains east to the Cascade foothills.

Over the 22‐year record, the annually averaged amplitudes show a significant negative trend (Kendall's tau = −0.54, *p*val < 0.0005), indicating decreasing summertime terrestrial low cloudiness (Figure 4b). EOF #2 declines are statistically significant in May (tau = −0.39, *p*val < 0.02) and also decline in June (tau = −0.30, *p*val = 0.06), July (tau = −0.25, *p*val = 0.11), and August (tau = −0.25, *p*val = 0.11).

### 3.4 EOF #3: Coastal Mode

EOF #3 explains 5% of PNW‐wide variance but is a locally important mode for the PNW coastal margin, explaining at least one third of within‐cell variance along the southern Oregon coast (white dots in Figure 4c). Over land, the dominant spatial loadings are restricted to only the lower elevation areas of the central Oregon coast immediately adjacent to the ocean that are plausibly exposed to marine influence. The structure of EOF #3, with dominant explained variance confined within 100 km of the coast in a southward widening wedge, is consistent with recognized links between summertime low clouds and the cool upwelled ocean waters of the northern California Current System (Byers, 1930; Checkley & Barth, 2009; Filonczuk et al., 1995; Huyer, 1976; Koračin et al., 2014; Leipper, 1994).

The coastal mode peaks in July but maintains a higher frequency of low clouds later into August and September than other regions (Figure 2). This midseason peak is further supported by cooccurring coastal airport records (Figure 3; e.g., Astoria, Quillayute, or North Bend). EOF #3 amplitudes have no significant trends over time.

### 3.5 EOF #4: Northern Coastal Mode

EOF #4 explains 3% of the total PNW variance but is locally important in the northern Pacific region of the domain, particularly off the coast of Victoria Island (white dots Figure 4d). EOF #4 does not exhibit any significant trends over time.

### 3.6 Declining Terrestrial Low Cloudiness

**Figure 5:**
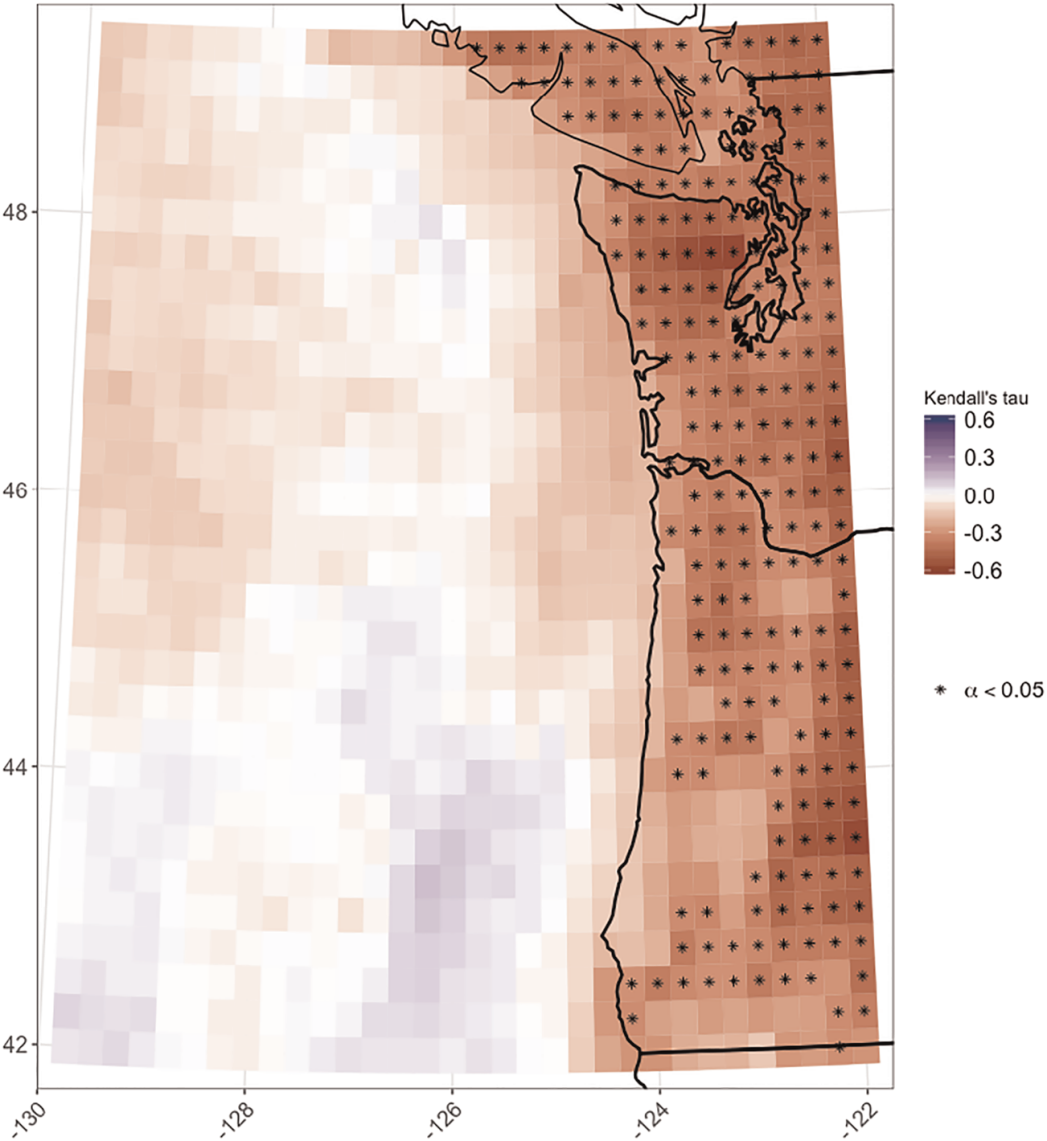
Kendall's tau statistic indicating the strength of the correlation between annual low cloud frequency and time, 1996–2017, for 0.24° × 0.24° GOES pixels.

A significant trend of decreasing monthly mean GOES‐derived low cloudiness was found for many terrestrial grid cells, independent of the EOF decomposition (Figure 5). However, although seasonal variations in low cloudiness at colocated GOES pixels and airports are similar, none of the airport data sets exhibit a statistically significant downward trend in the frequency of low clouds, though some airports did exhibit significant negative trends in total cloud frequency. Thus, the negative trend in satellite‐based estimates of low cloud frequency could be an artifact of the GOES low cloud detection algorithm, which was developed for marine and coastal areas, and may reflect a combination of low cloud frequency and all cloud frequency in inland areas. Additionally, there may be compounding factors due to the GOES algorithm specifically over land that enhance the decline without altering interannual or interdaily variability. One possibility might be identification of high elevation snow cover as low clouds. We expect that focusing specifically on summer and applying the 1,500‐m elevation mask should mitigate the snow problem, but snow does occur at some high elevation PNW locations into June. Recognizing these current methodological limitations, the observed declines should be interpreted cautiously pending further analysis.

## 4 Conclusions

### 4.1 Physical Context of Patterns and Trends

We show distinctive spatial and seasonal patterns in PNW low cloudiness. At the mean monthly cycle, we broadly identified four physically interpretable modes: oceanic, terrestrial highlands, coastal, and northern coastal. Over the Pacific Ocean (EOF #1), low clouds are controlled by large‐scale synoptic and climatological conditions, with stratus typically forming at the top of the moist marine atmospheric boundary layer stabilized by subsidence from above of warm, dry air creating a persistent inversion that traps the air below (Filonczuk et al., 1995; Koračin et al., 2014; Neiburger et al., 1961). During the Northern Hemisphere summer, subsidence combined with northwest surface winds and cold coastal upwelling over the Pacific create consistently frequent low cloudiness conditions (Filonczuk et al., 1995; Koračin et al., 2014). Our low cloud frequency maps show peak low cloud frequencies over the Pacific during midsummer when cloud‐creating conditions are strongest.

Low clouds over terrestrial highlands (EOF #2) are less frequent than marine or coastal clouds. Here, widespread intrusion of stratus‐forming marine air is likely blocked by the sharp topographic contrast of the Coast Range (Mass et al., 1986). Occasional onshore summertime surges of marine air, accompanied by stratus clouds, are a long recognized phenomenon for Oregon (Johnson & O'Brien, 1973) and Washington (Kinzebach, 1955; Mass, 1982; Mass et al., 1986). In these events, marine air penetrates lower elevation gaps in the Coast Range, reaching as far as the Western Cascades during strong events (Mass et al., 1986). Inland penetration of marine air and cloud cover is most common when the marine boundary layer is deep, allowing for cool and humid marine air to be transported over mountain barriers and develop a deep cloud layer that is difficult to burn off (Williams et al., 2015). Figure 2 shows higher frequencies in these low elevation areas (e.g., the Chehalis Gap, Columbia and Coquille River Valleys, and Strait of Juan de Fuca, identified in Figure 1). Conversely, least frequent cloudiness occurs at the highest elevation and farthest inland locations, where topographic relief and distance to ocean often prevents intrusion of low clouds.

We show satellite‐derived decline in low cloud frequency over time at inland locations and an increase over the ocean, a spatial trend broadly consistent with expectations for northward expansion of the dry, descending Hadley cell branch (e.g., Song et al., 2018; Tao et al., 2016); however, there is considerable uncertainty associated with definitively interpreting a relatively short (22‐year) record in the context of long‐term climate change. Additionally, due to possible limitations of the GOES low cloudiness algorithm over inland areas combined with a lack of consistently declining trends in corresponding airport records, the inland trend should be interpreted cautiously until further research is conducted.

Along the coast (EOF #3), low clouds are likely associated with the marine influence of cold sea surface temperatures from coastal upwelling (e.g., Checkley & Barth, 2009; Huyer, 1976; Koračin et al., 2014; Leipper, 1994), with inland extensions enhanced by temperature differences between warm terrestrial surface temperatures and cool marine temperatures (Mass et al., 1986). EOF #4 on the northern coast may also be related to sea surface temperatures but perhaps experiences an independent ocean response related to the coastal upwelling circulation along Vancouver Island (Bylhouwer et al., 2013). Fog over land is predominantly featured in EOFs #3 and #4, as incoming cool marine air interacts with summer‐warmed land over the coastal area known as the “fog belt”; the midsummer peak in low cloud frequency at all coastal airport locations (Figure 3) generally agrees with the expected seasonal timing of onshore surges of marine air known to generate coastal fog (Mass et al., 1986).

### 4.2 Social and Ecological Implications

Practically, low clouds have implications for ecosystem ecology (Fischer et al., 2009; Still et al., 2009), urban health (Clemesha et al., 2018), wildfire potential (Williams et al., 2018), and agriculture (Baguskas et al., 2018). Rapidly growing urban areas of the PNW, including Seattle, Vancouver, and Portland, lie in the EOF #2 mode where GOES‐derived low cloud frequency has been decreasing (Figures 4 and 5). Like the rest of the PNW, they are facing projected 21st century warming by as much 5°C by 2100 (Rupp et al., 2016). Urbanization also serves as a positive feedback, driving further warming and reductions in low cloud frequency (Williams et al., 2015). Possibly, we are already seeing feedback effects of the observed twentieth century PNW warming trend (0.8°C, Mote & Salathé, 2010) on declining terrestrial low cloud frequency, but this remains to be tested.

For ecosystems, vegetation function during rain‐scarce summers relies on low clouds. Supplemental moisture inputs and cloud shading can ameliorate forest drying and help maintain consistent water budgets and limit drying of burnable fuels. Cloudy conditions benefit tree physiology by decreasing vapor pressure deficits, thereby increasing canopy conductance (Wharton et al., 2012). Moreover, by increasing the fraction of diffused radiation, light can illuminate a greater portion of tall canopies, increasing overall canopy photosynthesis and its radiation use efficiency (Rastogi et al., 2018) and water use efficiency (Jiang et al., 2019). General circulation models used for predictive purposes still have difficulty simulating clouds with accuracy (Cesana & Waliser, 2016), and clouds are not included in downscaled climate data products (e.g., Abatzoglou & Brown 2011; Thrasher et al., 2013) or used explicitly to drive terrestrial biome simulation models (Wei et al., 2014).

In future work, our baseline assessment should be bolstered by continued monitoring of PNW low cloudiness. Our current 22‐year data series limits what can be learned from rigorous exploration of temporal variability, cyclicity, and climatic, ecological, and anthropogenic connections, which will be a critical avenue of future research to make robust projections of future changes and impacts of PNW low cloudiness.

## Data Availability

The original raw GOES variable format data were obtained from University of Wisconsin for 1996–2008 (https://www.ssec.wisc.edu/datacenter/goes-archive/) and for 2009 onward from NOAA, Comprehensive Large Array‐Data Stewardship System (CLASS) (www.class.noaa.gov).
